# Hydration Lubrication in Biomedical Applications: From Cartilage to Hydrogels

**DOI:** 10.1021/accountsmr.1c00219

**Published:** 2022-02-09

**Authors:** Weifeng Lin, Jacob Klein

**Affiliations:** Department of Molecular Chemistry and Materials Science, Weizmann Institute of Science, Rehovot 76100, Israel

## Abstract

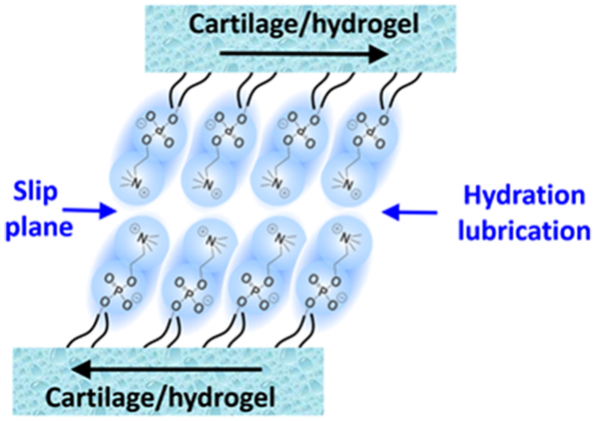

In the course of evolution, nature has achieved remarkably lubricated surfaces, with healthy articular cartilage in the major (synovial) joints being the prime example, that can last a lifetime as they slide past each other with ultralow friction (friction coefficient μ = the force to slide surfaces past each other/load compressing the surfaces < 0.01) under physiological pressures (up to 10 MPa or more)). Such properties are unmatched by any man-made materials. The precise mechanism of low friction between such sliding cartilage tissues, which is closely related to osteoarthritis (OA), the most widespread joint disease, affecting hundreds of millions worldwide, has been studied for nearly a century, but is still not fully understood. Traditionally, the roles of load bearing by interstitial fluid within the cartilage bulk and that of thin exuded fluid films at the interface between the sliding cartilage surfaces have been proposed as the main lubrication mechanism. More recent work, however, suggests that molecular boundary layers at the surfaces of articular cartilage and other tissues play a major role in their lubrication. In particular, in recent years hydration lubrication has emerged as a new paradigm for boundary lubrication in aqueous media based on subnanometer hydration shells which massively reduce frictional dissipation. The vectors of hydration lubrication include trapped hydrated ions, hydrated surfactants, biological macromolecules, biomimetic polymers, polyelectrolytes and polyzwitterionic brushes, and close-packed layers of phosphatidylcholine (PC) vesicles, all having in common the exposure of highly hydrated groups at the slip plane. Among them, vesicles (or bilayers) of PC lipids, which are the most widespread lipid class in mammals, are exceptionally efficient lubricating elements as a result of the high hydration of the phosphocholine headgroups they expose. Such lipids are ubiquitous in joints, leading to the proposal that macromolecular surface complexes exposing PC bilayers are responsible for the remarkable lubrication of cartilage. Cartilage, comprising ∼70% water, may be considered to be a complex biological hydrogel, and studying the frictional properties of hydrogels may thus provide new insights into its lubrication mechanisms, leading in turn to novel, highly lubricious hydrogels that may be used in a variety of biomedical and other applications. A better understanding of cartilage lubrication could moreover lead to better treatments for OA, for example, through intra-articular injections of appropriate lubricants or through the creation of low-friction hydrogels that may be used as tissue engineering scaffolds for diseased cartilage.

In this Account, we begin by introducing the concept and origin of hydration lubrication, extending from the seminal study of lubrication by hydrated simple ions to more complex systems. We then briefly review different modes of lubrication in synovial joints, focusing primarily on boundary lubrication. We consider modes of hydrogel lubrication and different kinds of such low-friction synthetic gels and then focus on cartilage-inspired, boundary-lubricated hydrogels. We conclude by discussing challenges and opportunities.

## 1 Introduction

The central functions of water in biology arise from the dipolar nature of its molecules and their ability to associate, i.e., to form hydrogen bonds, giving liquid water its unique properties and its role in the biochemical and biophysical processes that enable life.^1^ Our interest here is in its role in lubrication: how it modulates the frictional dissipation as two surfaces, whether synthetic or living tissue, slide past each other. In this context, the behavior of water under strong confinement, such as between two contacting surfaces, is of particular interest. One of the unique characteristics of water is its persistent fluidity, with a viscosity that remains comparable to that of bulk water when confined to thin films, down to a single molecular layer, an effect discovered only some two decades ago.^2^ In contrast, it is well established that the viscosity of nonassociative liquids (including most organic solvents and oils) diverges, and they become solidlike when they are confined by solid surfaces to films less than several molecular layers thick.^3^ This contrast under confinement may be viewed in terms of the different phase behavior of water and of nonassociating liquids: in both cases, the confining surfaces attract the confined liquids, which acts to densify them.^4^ In the case of water, however, densification suppresses its tendency to solidify^5^ (its solid phase, ice, is less dense than its liquid phase), while for most nonaqueous liquids it is the solid phase which is denser, leading to the confinement-induced solidification.^3^

**Figure 1:**
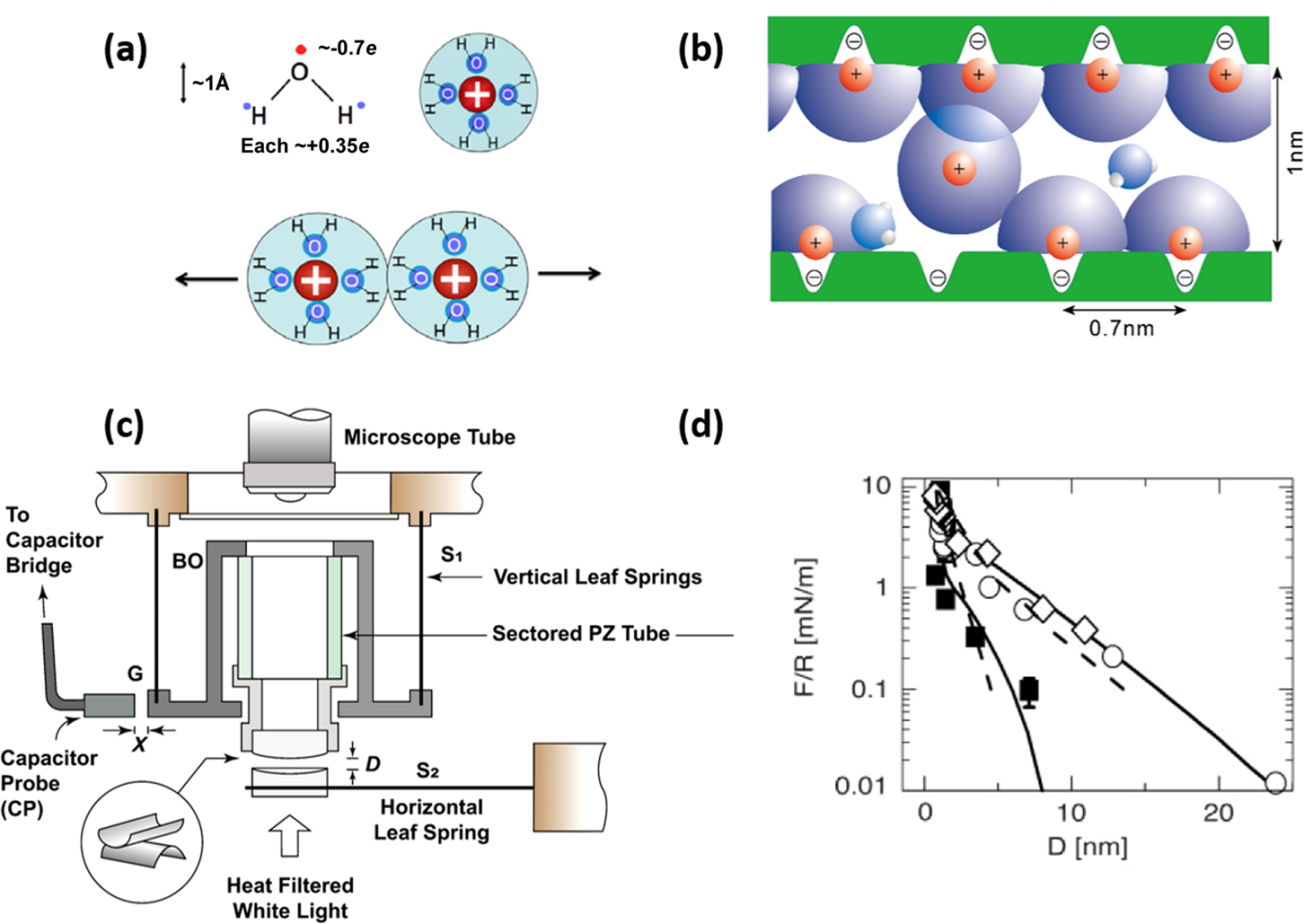
(a) Illustrating the large dipole moment of a water molecule due to the difference in the electronegativity of atoms (top left), a hydration shell of water molecules surrounding a positively charged ion (top right), and the hydration repulsion of steric origin due to the overlapped hydration layer of two similar charges (bottom).^6^ (b) Hydrated Na^+^ ions trapped between two negatively charged mica surfaces which are ca. 1 nm apart, where the spacing of the mica surface lattice sites is ca. 0.7 nm. This hydration layer can bear a large load without being squeezed out, while the molecular exchange rate between the water molecules in the hydration layer and bulk water, or other hydration layers, is very fast and ensures its fluidity.^7,8^ (c) Schematic illustration of a surface forces balance (SFB) for directly measuring normal and shear forces between two molecularly smooth, back-silvered mica surfaces.^9^ The absolute separation *D* (to ±2 Å) between two mica surfaces can be evaluated from multiple-beam interference, enabling normal forces to be measured by the bending of the horizontal leaf spring S_2_. Shear forces are determined via an air-gap capacitance probe, which measures the bending of the vertical leaf springs S_1_.^10^ (d) Normal force profiles between two mica surfaces across 0.007 ± 0.002 M (empty symbols) and 0.08 ± 0.01 M (solid symbols) of NaCl solution. Strong hydration repulsion due to an overlap of hydration shells on the trapped counterions was seen for *D* < ca. 2 nm during compression.^7^ (a) Reproduced with permission from ref (6). Copyright 2013 Springer-Verlag. (b and d) Reproduced with permission from ref (7). Copyright 2002 AAAS. (c) Reproduced with permission from ref (9). Copyright 2021 Wiley-VCH.

Water molecules are electrically neutral overall but have relatively large electric dipoles due to the difference between hydrogen atoms and oxygen atoms in their ability to attract electrons (as shown in Figure 1a). This results in water molecules being attracted to charges in aqueous solution (such as ions and zwitterions) by a dipole–charge interaction to form a loose layer around the charges, known as a hydration layer or shell (the water molecules within such shells comprise the water of hydration), within which the water dipoles are preferentially oriented toward the charge. This hydration layer reduces the self-energy (or Born-energy) of the enclosed charge substantially. This in turn means that it takes a large amount of energy (dehydration energy) to permanently remove a water molecule from the hydration layer and implies that the hydration layer can bear a large normal stress, for example, between two confining surfaces, without being squeezed out. At the same time, rapid exchange is possible between the water molecules in the hydration layer and adjacent free water molecules because that does not involve a permanent loss of water from the hydration shell (the exchange time can be as short as nanoseconds). This enables very rapid relaxation of the hydration shell, meaning that it has a high fluidity (as illustrated in Figure 1b). This combination of tenaciously attached hydration shells that are at the same time very fluid makes hydrated species excellent lubricating elements. They can sustain high loads (or stresses) without losing their hydration shells, and when sheared, these shells behave in a very fluid manner, i.e., exhibit low shear or frictional stress. The result is very low coefficients of friction (CoF or μ, defined as [force to slide]/[compressive load on surfaces]) up to high pressures. (Values of the normal stress required to fully remove the hydration layer from a Na^+^ ion in water have been estimated to be on the order of GPa.^6^) This mechanism, known as hydration lubrication, was first uncovered by Raviv et al.^7^ using a surface forces balance (SFB) (as shown in Figure 1c) to examine the friction between mica surfaces in relatively high concentration salt solution (ca. 0.1 M). Strong hydration repulsion due to an overlap of hydration shells on the trapped counterions was seen during compression (as shown in Figure 1d), and when the highly compressed surfaces slid past each other, the fluid hydration layers at the slip plane ensured a very low shear stress. In a subsequent study, Ma et al.^8^ directly determined the frictional dissipation due to viscous losses when such angstrom-thick hydration layers (surrounding Na^+^ ions) are sheared. The effective viscosity of the water in the hydration layers turns out to be ca. 200-fold larger than that of either bulk water or similarly confined water which is not in hydration shells. This effective viscosity of the subnanometer hydration layers is sufficiently low to account for the observed lubricating action of hydrated ions or zwitterions in a wide range of studies, as considered below.

**Figure 2:**
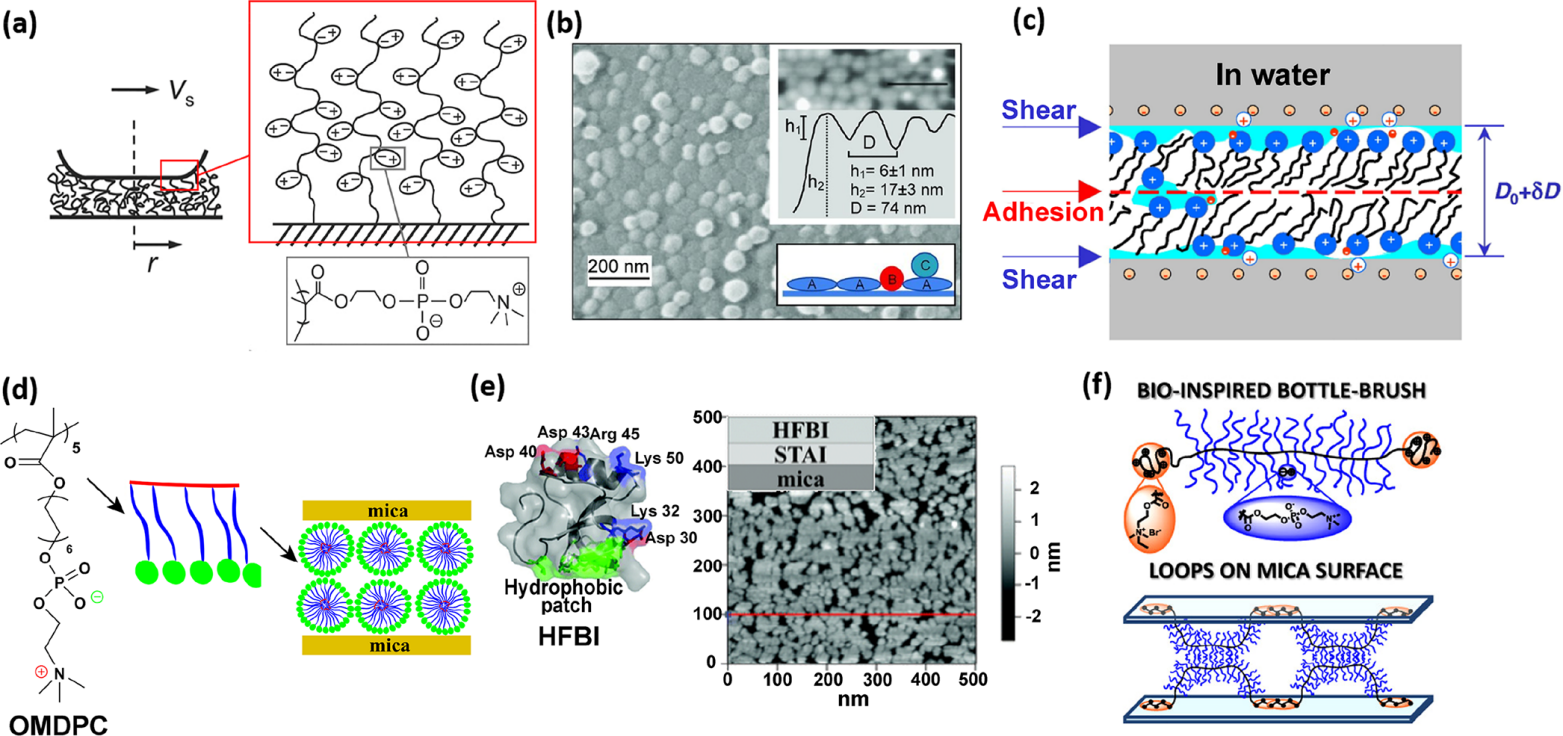
(a) PMPC polymer brush grafted directly from a mica surface can have a CoF of as low as 10^–4^ at pressures as high as 150 atm or more because of the intensive hydration of the strongly attached brushes consisting of phosphorylcholine-like monomers.^11,18^ (b) Hydrogenated soybean phosphatidylcholine (HSPC) liposomes are closely packed on a mica surface in the form of intact vesicles, which reduce the CoF down to μ ≈ 10^–4^–2 × 10^–5^ under a pressure of up to ca. 120 atm. This ultralow friction under physiologically high pressures is attributed to lubrication by the highly hydrated phosphocholine headgroups exposed at the interfaces, with close-packing structures on the sliding substrates and strong interactions between hydrophobic tails.^12^ (c) Swelling δ*D* is observed when surfactant-monolayer-coated mica surfaces in air (layer thickness of *D*_0_) are immersed in water (layer thickness of *D*_0_ + δ*D*), attributed to the hydration of the surface-attached headgroups. This hydration layer leads to a reduction in sliding friction via the hydration lubrication mechanism so that during surface shear under water the slip plane reverts from hydrophobic tails to the headgroup/mica interface.^13^ (d) Homo-oligomeric phosphocholinated micelles demonstrate excellent lubrication and are much more robust than single-tail phosphocholinated surfactants as a result of the greater energy required to remove a homo-oligomeric molecule from its micelle structure. The strong reduction in sliding friction can be attributed to hydration lubrication by the highly hydrated phosphocholine groups of the oligomer that are exposed at the interface between the close-packed micelles on the sliding substrates.^14^ (e) Schematic of the amphiphilic protein hydrophobin (HFBI) structure. The friction between HFBI proteins adsorbed hydrophobic-side down on hydrophobized mica (exposing their hydrated hydrophilic surfaces) is much lower than the friction between HFBI-coated hydrophilic surfaces (exposing the hydrophobic patches of the HFBI at the slip plane).^15^ (f) Schematic of the bottle-brush polymer inspired from the structure of lubricin. The lubricin-mimicking polymer consists of two cationic adhesive domains at its ends, and a central bottle-brush domain containing a flexible backbone modified with highly hydrated poly(2-methacryloyloxyethyl phosphorylcholine) (PMPC) brushes. CoF is as low as ∼10^–3^ under physiological pressures as a result of the exposure of the highly hydrated grafted PMPC brushes.^16^ (a) Reproduced with permission from ref (11). Copyright 2009 AAAS. (b) Reproduced with permission from ref (12). Copyright 2011 Wiley-VCH. (c) Reproduced with permission from ref (13). Copyright 2006 Nature Publishing Group. (d) Reproduced with permission from ref (14). Copyright 2020 American Chemical Society. (f) Reproduced with permission from ref (15). Copyright 2013 Royal Society of Chemistry. (e) Reproduced with permission from ref (16). Copyright 2014 American Chemical Society.

**Figure 3:**
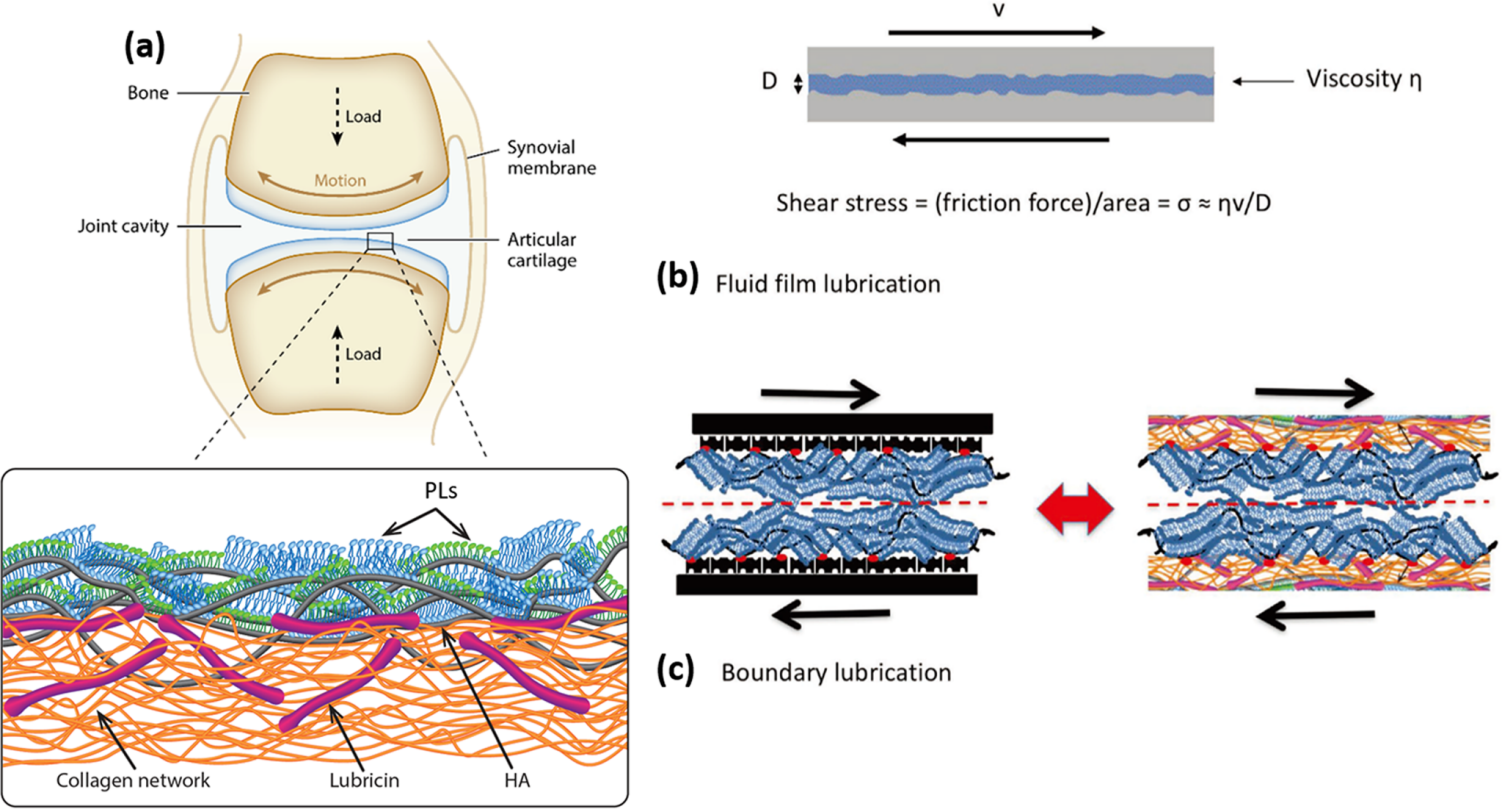
(a) Illustration of a synovial joint and its boundary layer consisting of linear hyaluronic acid (HA, gray), mucinous glycoprotein lubricin (purple), and phospholipids (monolayer, green; bilayer, blue) on a collagen network (orange). (b) In a fluid-film lubrication mode, a thin liquid layer separates the cartilage surfaces as they slide, with few asperity contacts, reducing the friction and wear markedly, and the shear stress σ_s_ may be written in the Newtonian form, σ_s_ = (shear rate) × (effective fluid viscosity).^27^ (c) In the boundary lubrication mode, frictional dissipation occurs at the interface between the opposing, contacting boundary layers and depends strongly on the detailed molecular structure at that interface, but it is largely independent of the substrates beneath the boundary layers. (a) Reproduced with permission from ref (27). Copyright 2016 Annual Review. (b and c) Reproduced with permission from ref (9). Copyright 2021 Wiley-VCH.

Hydration lubrication as described above, based on subnanometer hydration shells which massively reduce frictional dissipation, has emerged as a new paradigm for understanding friction and lubrication processes in aqueous media.^6^ The striking reduction of friction that this mechanism affords has been demonstrated in many systems, from the simple case above (Figure 1) where compressed, charged surfaces slide across trapped, hydrated counterions,^7,8^ to more complex situations. These include surface boundary layers composed of zwitterionic polymer brushes,^11^ phosphatidylcholine (PC) liposomes,^12^ hydrated surfactants,^13,14^ biological macromolecules,^15^ and biomimetic polymers^16^ (as illustrated in Figure 2). In particular, phosphocholine groups, whether as monomers on a polymer brush or as surface-attached PC liposomes or lipid bilayers, provide extremely efficient lubrication, down to μ = 10^–4^ or lower^12,17^ (as shown in Figure 2a,b). The origins of this have been extensively examined, particularly the low friction and the robustness of the surface-attached liposomes to high compression and shear. The low friction arises via the hydration lubrication mechanism acting at the slip plane between the outer surfaces of the opposing liposomes at the exposed, close-packed phosphocholine groups. These are known to be exceptionally highly hydrated, with some 15 ± 5 hydration water molecules per phosphocholine group, depending on the method by which this is determined.^11^ The robustness of the surface arrays of liposomes even when sliding at contact pressures of up to 100 atm or more (and for supported bilayers to slightly lower pressures) arises from the strong attraction between the acyl tails on the PC lipids.^12^ Indeed, it is clearly shown that longer tails (as in distearoylphosphatidylcholine, DSPC, with (C_18_)_2_ acyl tails) result in arrays that lubricate to much higher pressures than shorter tails (as in dimyristoylphosphatidylcholine, DMPC, with (C_14_)_2_ aryl tails).^19^ Much of the research on hydration lubrication is carried out on model substrates (atomically smooth mica or silicon surfaces) using nanotribometric techniques such as SFB^6^ and colloidally tipped atomic force microscopy.^20^ However, since boundary lubrication depends much less on the bulk substrate and much more on the contacting outer boundary layers (as illustrated in Figure 3(c)), the nanotribometric experimental results have a validity well beyond the model substrates used. Hydration lubrication has indeed been widely used to explain macroscopic experimental phenomena and biological lubrication, including cartilage lubrication,^9^ hydrogel lubrication,^21^ and machine lubrication.^22^

## 2 Cartilage Lubrication

Synovial joints, such as hips and knees, are remarkable biotribological systems, bearing most of the load of the human body while articulating, i.e., rotating, with very low friction when healthy; articular cartilage is the soft tissue coating the ends of joint bones where they are joined and articulate (as shown in Figure 3a). As noted, healthy articular cartilage presents the most slippery naturally occurring surface, with a CoF of as low as μ ≈ 0.001 up to physiologically high pressures. For example, the peak average walking pressure is ca. 5 MPa,^23^ and the highest pressures of up to around 20 MPa have been measured locally on the cartilage.^24^ Shear (frictional) stresses σ_s_ are felt by the cartilage surfaces as they slide past each other, decaying with depth into the cartilage. They may be written as σ_s_ = *μP*, where *P* is the local normal stress (which depends on body weight and activity). Such frictional stresses play an important dual role in cartilage degradation through both the usual wear-and-tear processes and, more subtly, through their effect on the chondrocyte cells (the only cell type in cartilage) embedded in its bulk. Chondrocytes are highly mechanosensitive: they require normal stresses to function optimally for cartilage homeostasis^25^ but upregulate catabolic genes (which produce cartilage-degrading enzymes) in response to shear stresses σ_s_.^26^ Thus, high friction, possibly initially induced by sports or accident traumas or by wear due to old age, and a consequently higher σ_s_, will lead to more cartilage-degrading enzymes. These in turn increase the friction as the cartilage surface degrades and so on in a self-reinforcing cycle,^9^ aggravated by the limited self-repair capability of the articular cartilage (which contains no blood vessels, lymphatic vessels, or nerves), eventually leading to joint degeneration and osteoarthritis (OA). An in-depth understanding of the lubrication mechanism of articular cartilage thus has significance both for the design of efficient biolubricants to treat early OA (for example by intra-articular injections) and for the design of hydrogels that might serve as synthetic cartilage for lesion repair or as scaffolds for cartilage tissue engineering.

The friction and lubrication of cartilage are usefully considered in terms of energy dissipation as surfaces move past one another, traditionally considered with respect to two main mechanisms (as illustrated in Figure 3b,c).^9,27^ In thin-fluid-film models, viscous energy dissipation, Δ*E*_viscous_ ≈ *A* ∫ [η_eff_ (d*x*/d*t*)/*D*] d*x*, takes place as the fluid film (trapped between the two cartilage surfaces) is sheared (*A* is the surface area, d*x*/d*t* is the sliding velocity, and *D* and η_eff_ are the thickness and effective viscosity of the fluid film, respectively). The thin-fluid-film models include hydrodynamic, elastohydrodynamic, squeeze-film, weeping, boosted lubrication, and other specific models.^27^ In a related mode, which does not require a thin fluid film to separate the surfaces, the interstitial fluid pressure within the cartilage is proposed to support most of the load on the joint (more than 90%).^28^ This is then proposed to reduce the effective contact pressure *P* between the opposing cartilage surfaces, resulting in low shear stresses and low sliding friction even at high loads.^29^ Experiments where cartilage explants slide across a glass countersurface and where the resulting sliding friction is shown to vary inversely with the simultaneously measured interstitial pressure in the cartilage have been carried out to provide direct evidence for this interstitial-fluid-pressurization lubrication mode.^30^ The other major mechanism for reducing frictional energy dissipation is boundary lubrication. In this mode, the opposing surfaces are in molecular contact, and the friction is mainly mediated by the intermolecular interactions and arises from irreversible processes (e.g., noncovalent bond breakage and re-formation) as the molecules slide past each other. The friction in this case is relatively insensitive to sliding speed *v*_s_ (in certain cases, it may vary logarithmically with *v*_s_(8)), in contrast to its linear (Newtonian) relationship with *v*_s_ in the fluid film mode. Linn et al.^31^ found that when hyaluronidase was added to the synovial fluid to reduce the viscosity of the synovial fluid, the CoF of the joint hardly changed. In contrast, if trypsin is added to the synovial fluid, it destroys the protein in the synovial fluid but has almost no effect on the synovial fluid viscosity whereas the CoF of the joint will be significantly increased, which shows that the joint is in a state of boundary lubrication. It is believed that cartilage lubrication is often in a so-called mixed regime, in which the main mechanisms, fluid film and boundary lubrication, are simultaneously active.^32^

**Figure 4:**
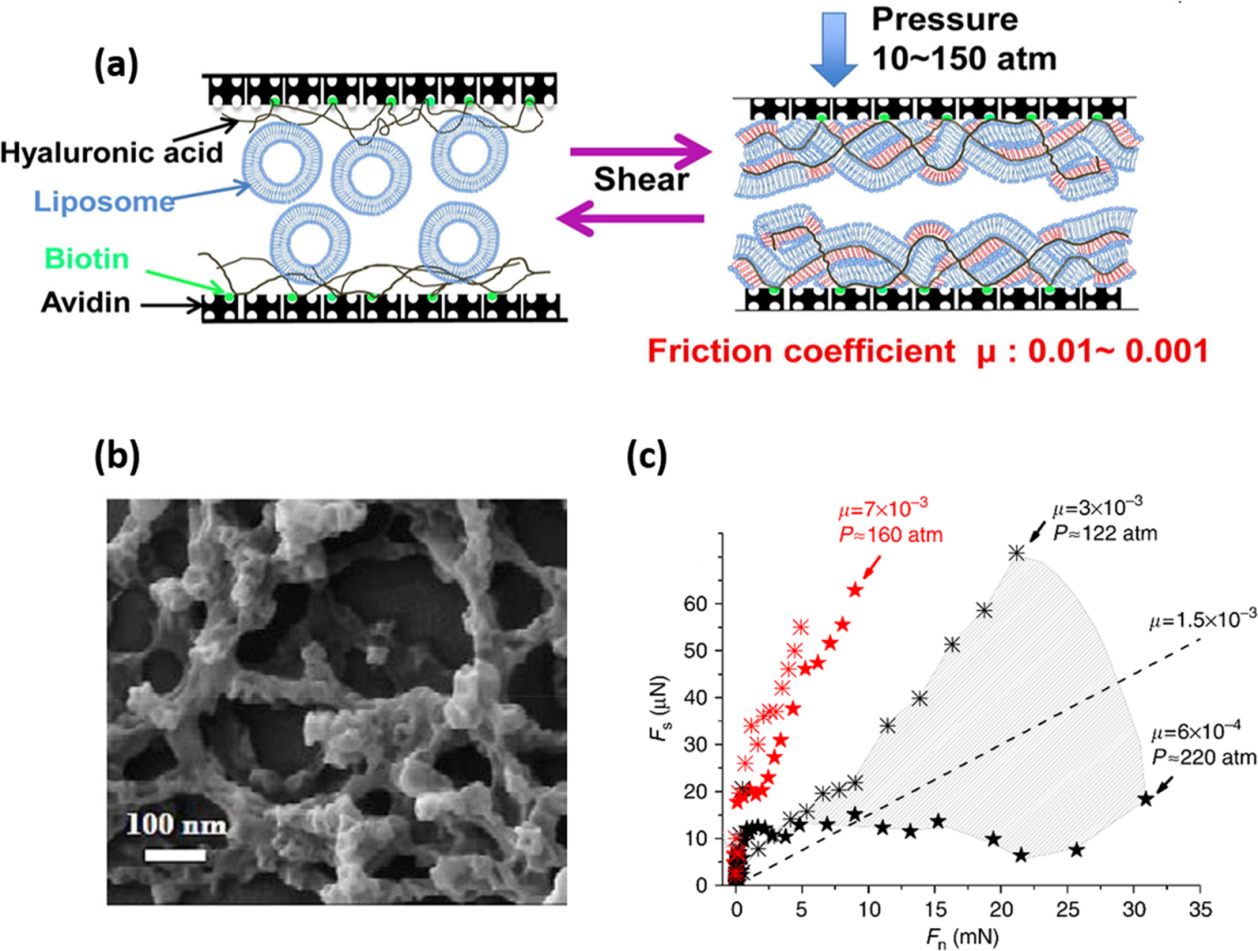
(a) Illustration of liposomes adsorbed on an HA-bearing surface, and the boundary layer structure proposed following compression and shear. (b) SEM image of HSPC liposomes attached on an HA-bearing mica surface.^37^ (c) Shear force *F*_s_ versus normal force *F*_n_ profiles between mica surfaces bearing surface layers of lipids complexed with HA, across purified water (black symbols) and across 150 mM KNO_3_ (red symbols).^36^ (a and b) Reproduced with permission from ref (36). Copyright 2017 Elsevier. (c) Reproduced with permission from ref (37). Copyright 2015 Nature Publishing Group.

In contrast to fluid film or interstitial-fluid-pressurization models, understanding cartilage boundary friction clearly requires a consideration of the detailed composition and structure of the biomolecules in the boundary layer. The main molecules that have been implicated in the lubricating cartilage boundary layers, either by themselves or in combination, are hyaluronic acid (HA, also called hyaluronan, a linear glycosaminoglycan), aggrecans (bottle-brush-like proteoglycans with mainly chondroitin sulfate side chains), lubricin (a mucinous glycoprotein), and phospholipids (PLs), as well as others.^27^ Direct measurements of normal and shear forces between surfaces coated with the above biomolecules have been made using a variety of methods, including SFB approaches and friction force microscopy (FFM) techniques,^12,33^ as well as macroscopic tribometry and torque-based approaches (rheometer).^34,35^ However, none of these biomolecules acting as a boundary layer can, by themselves, explain the low friction of the cartilage surface at the high-pressure characteristic of the major joints. An elucidation of the lubricating boundary layer on healthy cartilage thus remains a major challenge. Clues are provided by recent SFB work in our group,^36,37^ indicating that PC lipids can complex with surface-attached HA to provide boundary structures that result in CoF values of as low as μ ≈ 0.001 at pressures *P* above 100 atm, as shown in Figure 4. These results reveal the synergistic action (on a model, noncartilage surface) of at least two of the main components to which cartilage boundary lubrication has been attributed, namely, HA and phospholipids, to provide a lubricity similar to that of healthy cartilage (μ ≈ 0.001 at *P* up to 100 atm or more). Such a synergy directly demonstrates the role of hydration lubrication because slip occurs at the highly hydrated phosphocholine headgroup interface exposed by the HA-PC surface complex: this suggests a way forward to a better understanding of how cartilage boundary layers lubricate.

Some recent macroscopic tribological studies also support the centrality of a boundary lubrication mechanism. Murakami et al.^38^ measured friction between a cartilage explant sample sliding under sustained compression across a glass surface. When they used pure saline as the immersion medium, they observed that the CoF increased from its initial low value (μ ≈ 0.01) with cartilage compression time as the interstitial fluid pressure decreased, in line with earlier studies.^30^ However, when HA, albumin, and DPPC were added to the saline, Murakami et al.^38^ found very little change with compression time and the CoF retained its low value (μ ≈ 0.01), despite the fact that the interstitial fluid pressure decreased. This implies that boundary lubrication, which is largely independent of any interstitial fluid pressure, may play a major role in reducing cartilage friction. In a more recent study, again using cartilage explants sliding under compression across glass, Hilser et al.^39^ showed that the CoF becomes essentially independent of compression time when using a lubricant composed of HA and lipids in a saline medium, while it increases with time, as previously seen,^30^ when using saline alone. Both of these studies demonstrate that lubricants comprising HA and lipids may account for the low friction of cartilage without the need for the interstitial-fluid-pressurization mechanism, in line with the SFB results for hydration boundary lubrication by these components.^36^ Such insight into the lubrication of cartilage, which may be viewed as a complex biological hydrogel in view of its large (∼70%) water content, suggest that synthetic hydrogels may also be lubricated by similar approaches. Efficient lubrication of hydrogels may thus in turn shed light on cartilage lubrication as well as having clear benefits when using hydrogels for cartilage repair^9^ and for their better performance in biomedical applications such as the low-friction coating of catheters or in contact lenses.^40^

## 3 Lubrication Mechanism of Hydrogels

Hydrogels are water-permeated 3D polymer networks with a high-water content and a low modulus resembling those of biological tissues, rendering them the material of choice for many applications in the field of biomedical devices and regenerative medicine.^41,42^ Approaches to repairing osteoarthritic cartilage lesions have included using hydrogel-based “artificial cartilage” to fill up (and thus “repair”) the lesions^32^ or for the tissue engineering of cartilage, where cell-incorporating hydrogel scaffolds are glued into lesions.^43,44^ In both cases, it is important that the hydrogel surfaces be sufficiently well lubricated in order that articulation at the high pressures of the joints does not shear them off (delamination) during repeated contact, deformation, and sliding.^43^ Similar to cartilage lubrication, the mechanisms of hydrogel lubrication can be divided into two main categories: fluid film lubrication and boundary lubrication.^45^ Fluid flow dynamics within the hydrogels, where the flow results in viscous dissipation, and their elastic deformation, play important roles in this.^46−49^ The physicochemical attraction or repulsion between the hydrogel network polymers and the substrate, or between the polymers on opposing different hydrogels when sliding past each other, will likewise strongly affect the friction. Repulsion between the chains and the sliding countersurface tends to draw fluid into the interface between them, while adsorption drives the fluid out, leading to more solidlike friction. On the other hand, polymers in the hydrogel network may also be highly hydrated even when attracted to the countersurface, resulting in a hydrated boundary layer during sliding.

Gong et al.^50^ developed the repulsion–adsorption model, which made a significant contribution to understanding the mechanism of hydrogel friction. In this model, when the interaction between the hydrogel and the substrate is repulsive, friction reduction mainly arises from lubrication by the fluid layer trapped between the gel and the substrate. Such essentially hydrodynamic lubrication shows a close-to-linear dependence of the friction on the sliding velocity, as expected for viscous shear stress in sheared Newtonian fluids. On the other hand, if the network polymers have a propensity to adsorb on the substrate, then another mechanism plays a role in addition to the viscous force exhibited by the lubricating fluid layer: this is the elastic deformation force required to detach the adsorbing polymers from the substrate during sliding. (Such detachment is dissipative because of its irreversible nature.)

**Figure 5:**
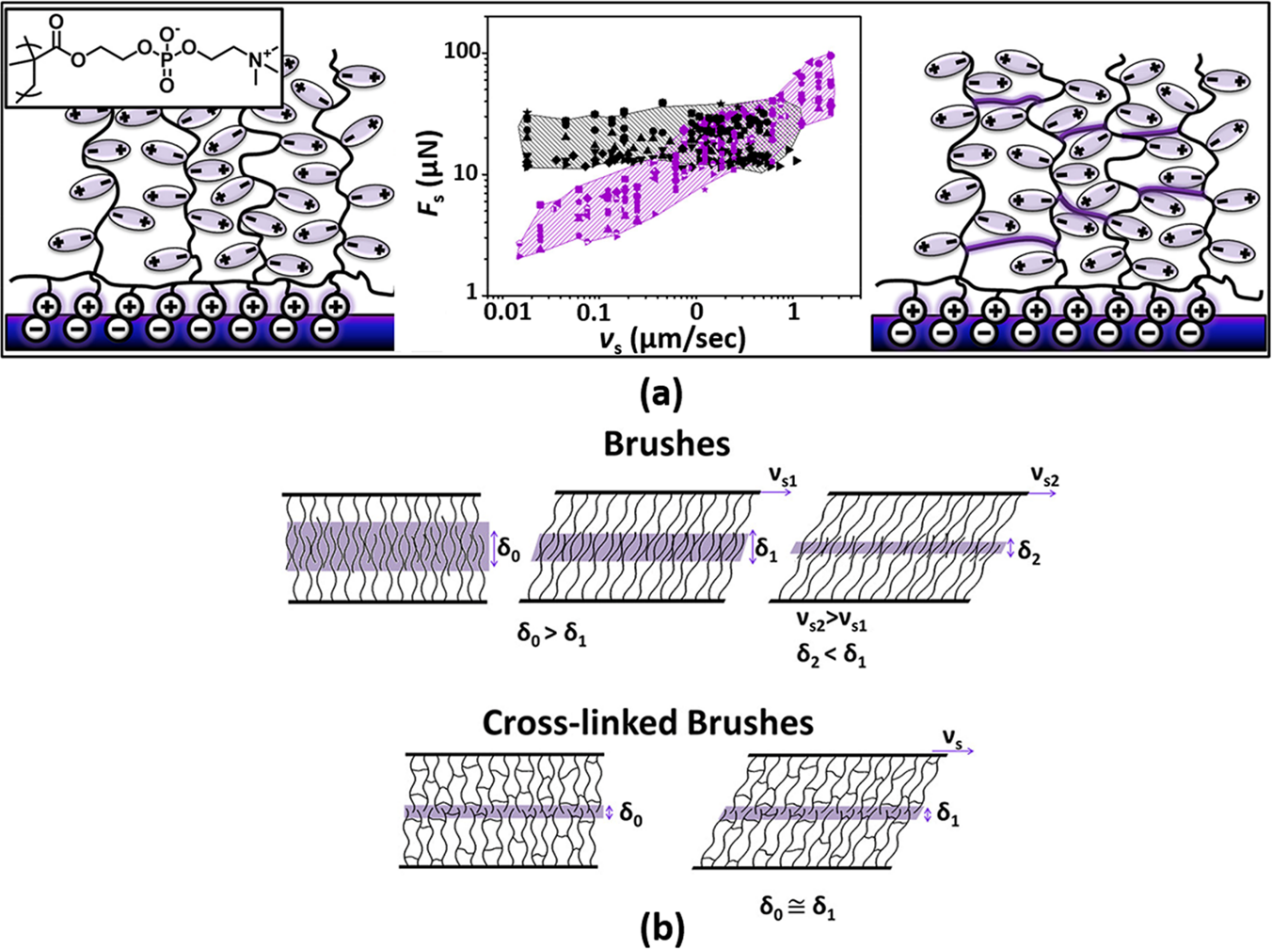
Illustration of the difference between lubrication by PMPC brushes grown from a mica surface ((a) left-hand cartoon) and the same brushes once they are internally cross-linked to form a thin gel-like layer ((a) right-hand cartoon). In both cases, the friction *F*_s_ is low, mediated by the hydration lubrication mechanism at the highly hydrated phosphocholine groups, but the dependence on sliding velocity *v*_s_ is very different ((a) center panel, μ ≈ 10^–4^ for brushes and μ ≈ 10^–3^–10^–4^ depending on the *v*_s_ for cross-linked brushes). (a) Comparison of the friction *F*_s_ versus sliding velocity *v*_s_ between PMPC brushes (black symbols) and PMPC hydrogel-like layers (purple symbols). (b) Schematic illustrations of the interpenetration zone (shaded area) between two compressed layers of either linear brushes or hydrogel-like cross-linked brushes. In the former case, the thinner interpenetration region at higher sliding velocities offsets the higher energy dissipation at such velocities, leading to very weakly velocity-dependent shear force (black data points in (a)). Reproduced with permission from ref (21). Copyright 2017 American Chemical Society.

Hydration lubrication based on hydrated boundary species (noted in previous sections) may also be used to reduce hydrogel friction. Iuster et al.^21^ constructed layers of internally cross-linked and non-cross-linked PMPC brushes, some tens of nanometers thick, on mica substrates, where such internally cross-linked brushes have the structure of a thin hydrogel layer, and measured the normal and shear interactions between them using an SFB. Although both brushes (μ ≈ 10^–4^) and cross-linked brushes (μ ≈ 10^–3^–10^–4^ depending on the *v*_s_) exhibit low CoF values (Figure 5a), attributed to hydration lubrication between the highly hydrated phosphorylcholine groups on the brushes, there was a significant difference in the velocity dependence of the friction. Friction *F*_s_ between the non-cross-linked polymer brushes shows a very weak dependence on *v*_s_ because of the self-regulating interpenetration of the opposing chains (as illustrated in Figure 5b). In contrast, friction between the hydrogel-like layers (intra-cross-linked brushes) shows *F*_s_ ∝ *v*_s_^α^, where the exponent α ≈ 0.5 indicates behavior arising from the essentially constant interpenetration of the cross-linked brushes imposed by the cross-linkers (so that no self-regulation is possible).

## 4 Low-Friction Hydrogels

Low-friction hydrogels have clear applications in a variety of biomedical areas where lubrication is at a premium (e.g., soft contact lenses, catheter coatings, and scaffolds for cells in tissue engineering) and thus are a promising topic in soft matter research. Their behavior may also cast light on the friction mechanism of biological hydrogels, such as the articular cartilage described earlier. Different approaches have been taken to prepare low-friction hydrogels.

### 4.1 Linear Polymer/Loosely Cross-Linked Polymer-Modified Hydrogels

**Figure 6:**
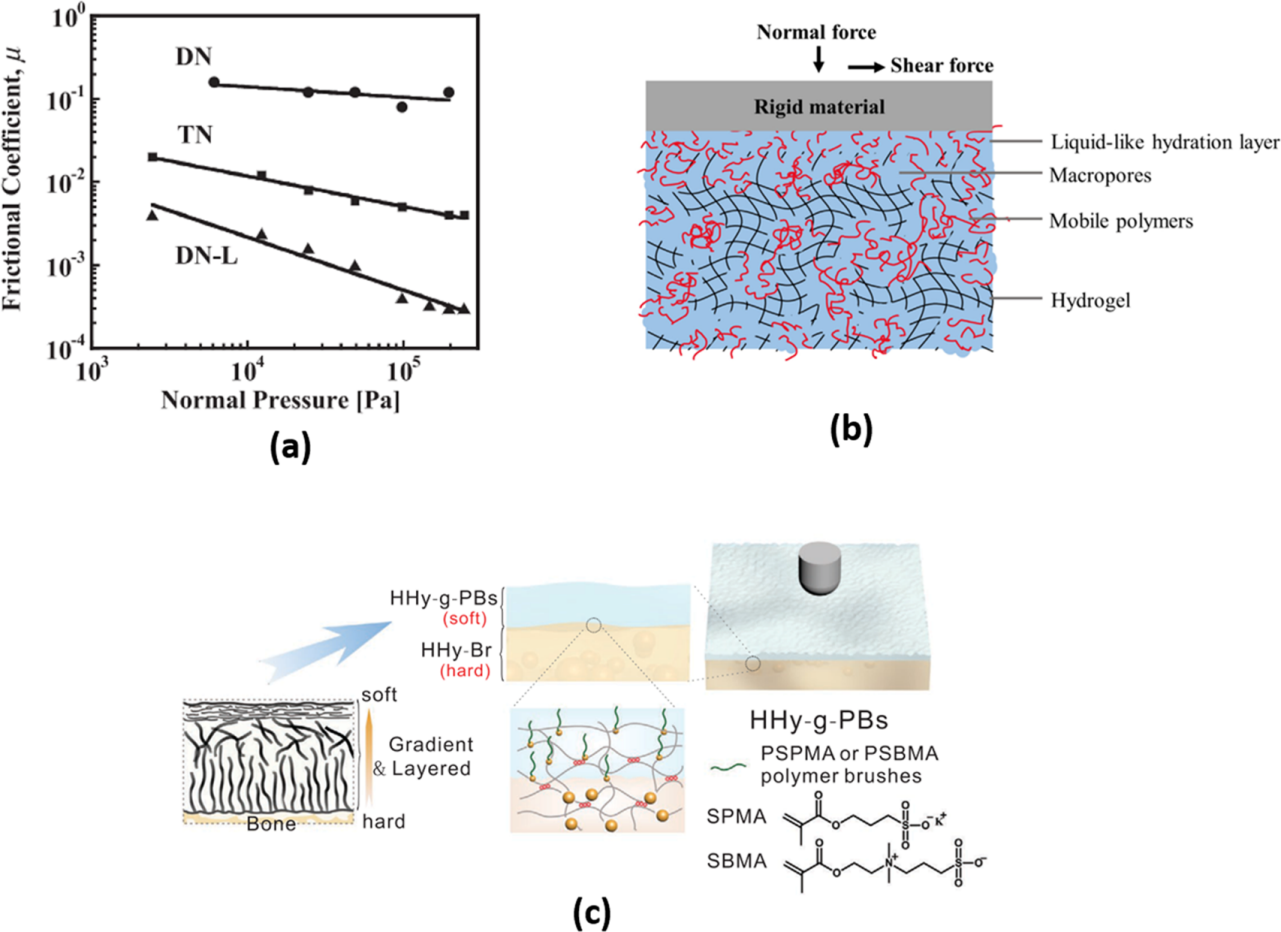
(a) CoF values of different hydrogels (DN, TN, and DN-L) against a glass plate in pure water.^51^ (b) A polymer-filled microporous hydrogel reduces friction when sliding against a rigid counter face under normal and shear forces.^57^ (c) Schematic illustration of the fabrication procedures of cartilage-mimicking PSPMA or PSBMA brush-grafted hydrogels.^58^ (a) Reproduced with permission from ref (51). Copyright 2011 Wiley-VCH. (b) Reproduced with permission from ref (57). Copyright 2020, Elsevier. (c) Reproduced with permission from ref (58). Copyright 2020 Wiley-VCH.

Double network (DN) hydrogels are a class of tough gels composed of a relatively rigid polyelectrolyte as the primary network (rigid due to strong repulsion between charged monomers and higher cross-linking) and an interpenetrating, loosely cross-linked, flexible neutral polymer as the secondary network. However, DN hydrogels have large CoF values, μ ≈ 10^–1^. Kaneko et al.^51^ prepared triple-network (TN) hydrogels by incorporating a third, weakly cross-linked network (negatively charged) as well as DN hydrogels incorporating linear, non-cross-linked, negatively charged chains (DN-L) of the same polymers as the primary network of the original DN hydrogel. The CoF values of such TN hydrogels (μ ≈ 10^–3^–10^–4^) and DN-L (10^–2^–10^–3^) hydrogels are significantly lower than that of DN hydrogels at the same sliding velocity (as shown in Figure 6a). These linear polymer chains (in DN-Ls) and loosely cross-linked polymers (in TNs) may act to lower the friction by providing a more fluid polymer surface phase. An interesting effect was observed regarding the substrate on which the hydrogels were prepared. Hydrogel surfaces in contact with a hydrophobic surface during their preparation exhibited dangling polymer moieties, thereby providing a more fluid and thus a better lubricating surface layer. Their friction coefficient was some 1 to 2 orders of magnitude lower than for hydrogel surfaces that had been in contact with hydrophilic substrates during their preparation for otherwise chemically identical gels.^52−54^ In a recent study,^55^ a biomimetic lubricant, loosely cross-linked PMPC, was introduced into a DN hydrogel, reducing its friction coefficient by up to ca. 50% (over a certain sliding-velocity range), an effect attributed to boundary lubrication. This approach was also applied with biological tissue (degraded cartilage) to improve its wear resistance.^56^

### 4.2 Polymer-Filled Hydrogels

To emulate the porous structure of cartilage, where the pores are permeated by different mobile biopolymers, Mu et al.^57^ fabricated a polymer-filled macroporous hydrogel (as shown schematically in Figure 6b) by soaking the macroporous gel in the polymer solution. The resulting CoF was one order of magnitude lower than that of the macroporous hydrogel without polymers as a result of liquid-like lubrication by the incorporated mobile chains. To control the lubrication in a reversible manner, Wang et al.^59^ designed a supramolecular hydrogel by incorporating into hydrogels a noncovalently cross-linked supramolecular network (α-cyclodextrin (α-CD)/poly(ethylene glycol) (PEG)) together with a competitive photoresponsive guest. Irradiation by UV and visible light enables association and dissociation between α-CD and the guest molecules. This in turn releases or recombines the PEG (in other words, causes the appearance or disappearance of a thin water-filled polymer layer) on the hydrogel surface. The resulting sliding CoF under UV of the hydrogel is ∼0.01, which is ca. 3-fold lower than that of the hydrogels under visible light.

### 4.3 Polymer-Brush-Grafted Hydrogels

To mimic the near-surface structure and presumed surface lubrication mechanism of articular cartilage, where a softer exposed layer is attached to a harder matrix beneath, Rong et al.^58^ grafted very thick (ca. tens of micrometers) polymer brushes on the surface of a stiff hydrogel. This hydrogel can maintain low friction (∼0.01–0.02) under high contact pressure (∼10 MPa): the top, thick polymer brushes provide efficient lubrication, and the bottom, stiff hydrogel gives the load-bearing capability (as indicated in Figure 6c).

### 4.4 Lipid-Based Boundary-Lubricated Hydrogels

**Figure 7:**
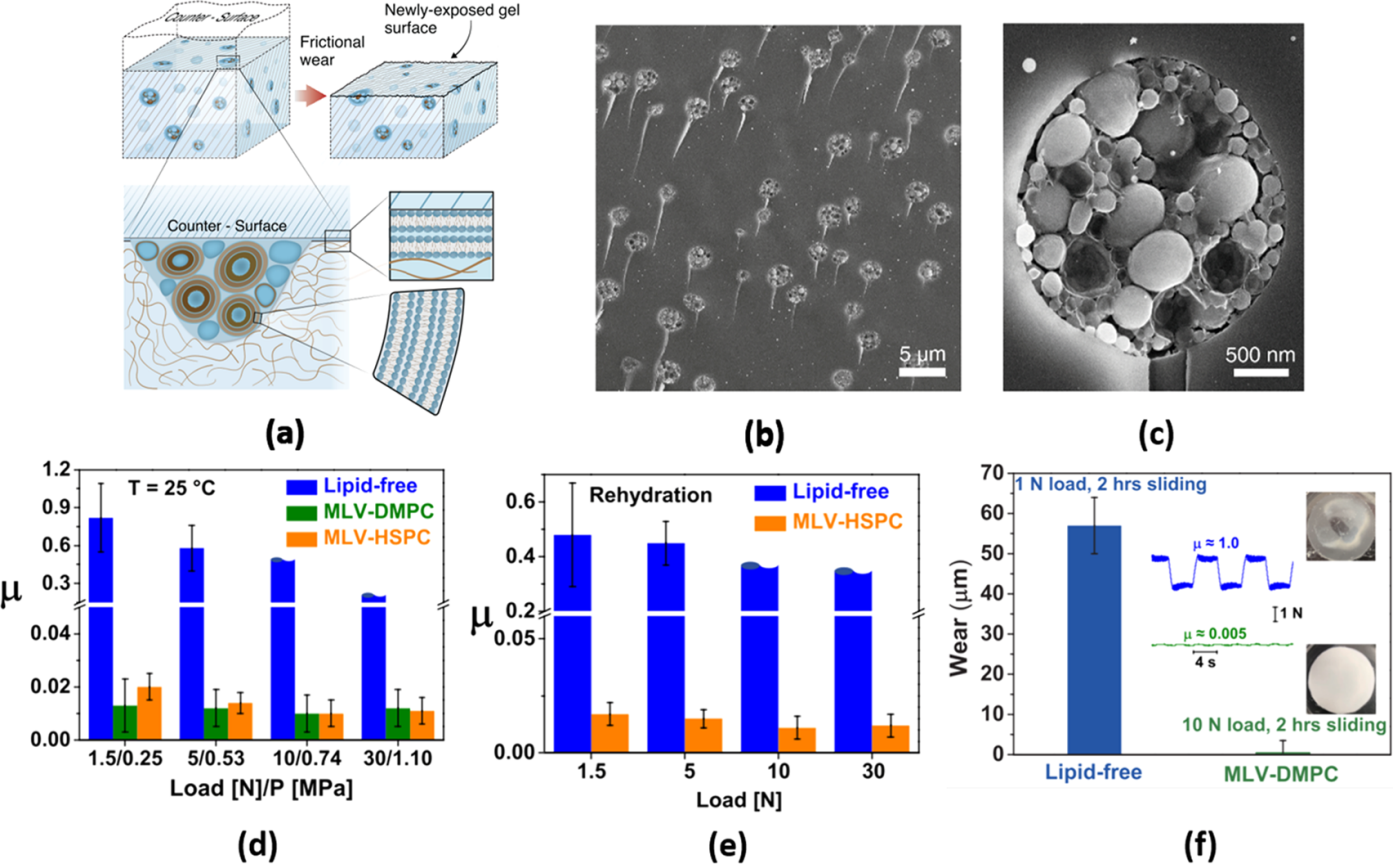
(a) Schematic diagram of a self-lubricating hydrogel containing phospholipids. (b) Freeze-fracture surface of the hydrogel containing DMPC vesicles by cryo-SEM, showing the microreservoirs transected by the surface. (c) Single microreservoir from (b) at higher magnification. (d) CoF of the hydrogel under different pressures. (e) CoF values of the lipid-free and HSPC-incorporating hydrogels under different pressures after rehydration, maintaining the characteristic self-lubricating ability for the latter. (f) Comparison of wear conditions of lipid-free and DMPC-incorporating gels. Reproduced with permission from ref (60). Copyright AAAS.

As discussed above, a significant part of the remarkable lubrication of cartilage is attributed to highly hydrated, lipid-exposing boundary layers.^9^ In living cartilage, it is the embedded chondrocyte cells which synthesize the lipids (as well as all other components of the cartilage) continuously and enable such lipids to accumulate at the surfaces. Inspired by this, hydrogels incorporating liposomes were prepared,^60^ with the liposomes aggregated within microreservoirs, which could continuously release lipids to create lipid-exposing boundary layers (as indicated in Figure 7). Such hydrogels indeed exhibited sustained, low friction (μ ≈ 0.005–0.02), and exceptionally low wear compared to the same but lipid-free hydrogel, under a range of conditions, and fluorescent labeling showed that this was due to lipid layers on the gel surface. At the same time, the incorporated lipids, at ca. 1% or even lower volume fraction, had little effect on the mechanical properties of the hydrogel. Lin et al.^60^ showed that sliding is the key driving force for the formation of a lipid layer on the surface of the hydrogel, by extracting lipids from the liposome microreservoirs adjacent to and transected by the hydrogel/counterface interface. Both the weak dependence of friction on the sliding speed and the measured thickness of the interfacial boundary layer formed by the phospholipids between the friction pair show that the striking lubricity arises from hydration lubrication acting at the slip plane between the lipid-exposing boundary layers on the two sliding surfaces. Compared to the incorporation of lipids into the hydrogel, the lubrication effect is greatly reduced if the liposome is simply added externally as a lubricating solution because it is difficult for the lipids in the external solution to enter the contact region between the gel and countersurface. Remarkably, the lubricity of the lipid-incorporating hydrogel is maintained when it is completely dried and then rehydrated by exposure to water; this property may be important for the long-term storage and application of the hydrogel under field conditions.

## 5 Conclusions and Outlook

Our initial account of hydration lubrication was extended to the surface of cartilage, a complex biohydrogel, where such a low friction mechanism is believed to apply, and from that to lubrication of and by synthetic hydrogels. We conclude by considering a number of outstanding challenges and opportunities in this area. These include the following:

1. Reducing friction between articular cartilage surfaces remains an important problem because of the intimate connection with osteoarthritis (OA). Of the main modes to which the lubrication of healthy cartilage has been attributed, (a) lubrication arising from load-bearing interstitial fluid pressurization and (b) boundary lubrication, there is little one can do to treat fluid pressurization in joints. However, boundary lubrication can indeed be modified, and suitable vectors that can form efficiently lubricating boundary layers are thus at a premium. The use of liposomes (in the form of small unilamellar or larger multilamellar vesicles, possibly in combination with other macromolecules such as HA and possibly lubricin) for intra-articular injections is an area ripe for exploitation. Because liposomes are also widely used as drug carriers, such lubrication vectors could be used at the same time for the controlled release of anti-inflammatory or pain-reducing drugs, which could be an effective treatment, both palliative and disease-suppressing, for OA.
2. Further on the topic of articular cartilage, lubricants, particularly liposomes, functionalized with targeting moieties which can tightly bind to the cartilage surface should be designed. Such a system will avoid the loss of lubricants within the circulatory system and out of the synovial membrane and maintain boundary layers on the cartilage surface for extended periods.
3. A related area to be explored concerns the preparation of hydrogels with desirable mechanical properties (strong and tough) and high water content (up to 70–80%), such as double-network hydrogels, incorporating highly hydrated lubrication vectors that would provide a reservoir to replenish boundary layers at the gel surface. Such layers may act through the hydration lubrication mechanism, resulting in extremely low friction and low wear and, unlike surface-applied or surface grown layers, would be replenished as they wear, thereby providing long-term lubricity. An associated and challenging problem to be resolved concerns the effective adherence of the lubricant-incorporating hydrogels in their wet state either to biological tissues such as cartilage lesions or to biomedical devices (e.g., catheters or endoscopes) whose use calls for lubricated surfaces.
4. Biocompatibility is a key requirement for the biological application of lubricants, whether directly on biological tissues (e.g., in eye drops for dry-eye syndrome) or for lubricated hydrogel coatings for biomedical devices. The use of liposomes should be further explored in this context because, almost uniquely, they are both naturally biocompatible because they are ubiquitous in living organisms and they can assemble to provide extremely lubricious boundary layers via the hydration lubrication mechanism. Recent indications suggest that examining the ability of liposomes to form more robust and efficient boundary layers in combination with other biomacromolecules is another direction that should be investigated.
